# Less is more: information needs, information wants, and what makes causal models useful

**DOI:** 10.1186/s41235-023-00509-7

**Published:** 2023-08-30

**Authors:** Samantha Kleinberg, Jessecae K. Marsh

**Affiliations:** 1 Computer Science Department, Stevens Institute of Technology, Hoboken, NJ USA; 2 Department of Psychology, Lehigh University, Bethlehem, PA USA

**Keywords:** Causal models, Decision-making, Information complexity

## Abstract

Each day people make decisions about complex topics such as health and personal finances. Causal models of these domains have been created to aid decisions, but the resulting models are often complex and it is not known whether people can use them successfully. We investigate the trade-off between simplicity and complexity in decision making, testing diagrams tailored to target choices (Experiments 1 and 2), and with relevant causal paths highlighted (Experiment 3), finding that simplicity or directing attention to simple causal paths leads to better decisions. We test the boundaries of this effect (Experiment 4), finding that including a small amount of information beyond that related to the target answer has a detrimental effect. Finally, we examine whether people know what information they need (Experiment 5). We find that simple, targeted, information still leads to the best decisions, while participants who believe they do not need information or seek out the most complex information performed worse.

## Significance statement

Computational methods and domain experts aim to create comprehensive models that can guide people’s decisions on topics such as health or personal finance. These models strive to include as many factors as possible (e.g., every known factor affecting body weight) while still being applicable to specific decisions (e.g., what a person should eat today). While these models can be considered complete, are they useful? Work in psychology has found that people prefer simple explanations in some situations, while preferring complex explanations in certain domains. An open question remains: does the amount of information people want match the amount of information they need to make a decision? We find that presenting people with simplified models leads to the best decisions, compared to complex models or no information. Second, we find that people’s information desires do not always match their information needs. Regardless of what models people chose, simple models were still the most helpful. This work has significant implications for how to create useful causal models and how information should be given to decision makers. We find that content needs to be finely tailored to the decision at hand.

## Introduction

We make many decisions on topics we do not fully understand. We choose how to manage our finances, careers, health, and the environment around us, and often do so with incomplete knowledge about how these complex systems work. Some decisions, like what to eat for breakfast, may have short-term consequences (that add up over time), while others like what house to buy may impact our lives for decades to come. As a result, many of us seek out information before making these choices. Even when we do not actively seek out information, we often are bombarded with guidance on topics like health, about which we make daily decisions. Whether implicit or explicit, the guidelines we receive about these topics rely on causal relationships. We are told to eat more of a specific food or to go for a daily walk because these actions can bring about desirable outcomes. Yet the information we receive is often piecemeal guidance about specific causal relationships (e.g., stress causes weight gain) rather than a complete causal model (e.g., all of the factors that result in weight gain, and that cause or result from stress). Acting on one relationship without understanding the broader causal context in which it operates can produce unanticipated side effects. For example, while running may lead to endorphins that reduce stress, running can also yield an injury that leads to stress. Having a complete causal model means we can identify and potentially prevent such undesirable side effects.

**Fig. 1:**
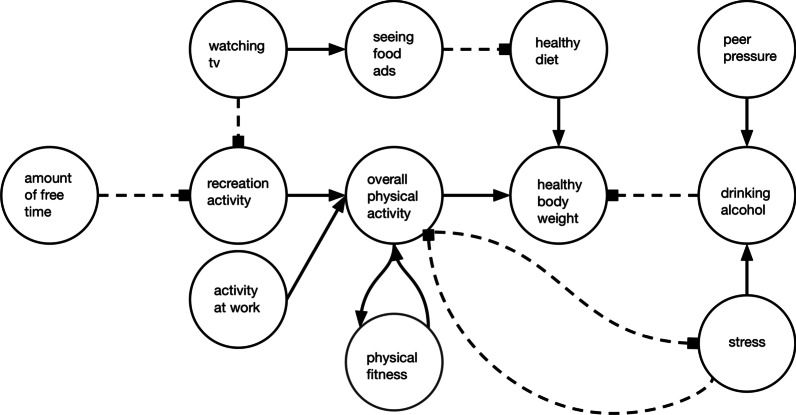
Complex causal diagram on managing bodyweight

Methods from computer science have made it possible to take data and learn all causes of a phenomenon (Kleinberg, 2012; Pearl, 2000). For some topics, researchers and domain experts have manually created models showing how factors are interrelated. For example, Fig. 1 depicts a simplified version of the Obesity System Atlas developed by the UK government. The full diagram encompasses psychological, social, and other factors influencing obesity and has over 100 nodes with many causal connections between them.^1^ Similarly detailed models can be found in other domains, including a model of American military strategy in Afghanistan that was derided in the news for its overwhelming complexity^2^ and a model of factors influencing mental health (Kinderman et al., 2013). The complexity of causal diagrams has been used to argue against policies, such as when the US Congress’s Joint Economic Committee minority mapped what they called the “bewildering complexity” of the Affordable Care Act to argue that rather than making decisions with their doctors, individuals would be trapped in a “never ending web of bureaucracy.”^3^ Given the complexity of real-world situations, those who truly want to use these complex models to help people make decisions must choose between providing the fullest accounting of the causal structure (that may overwhelm decision makers) and providing simplified versions (that leave out relevant detail). Despite its importance, little is known about how this trade-off between completeness and understandability affects decision making.

Even though real-world systems and their causal models can be overwhelmingly complex, people may appreciate the complete picture such models present. Korman and Khemlani (2020) found that people perceive a single integrated causal model as more complete than a system that is split into multiple models. While research has shown a preference for simpler explanations (Lombrozo, 2007, 2016), such preferences depend on both what type of system is being explained (Johnson et al., 2019) and its perceived complexity (Lim and Oppenheimer, 2020; Zemla et al., 2017). Taken together, this work suggests that if people are presented with a complex causal model diagram, they may judge it as more complete than a set of models each showing a different component of a system.

While complexity may have advantages such as enabling more complete models, individual preferences may also play a role in how much complexity people desire in causal diagrams. Within consumer choice, Fernbach et al. (2013) found that while some individuals wanted detailed explanations of how a product worked, others preferred less information. These preferences influenced hypothetical purchase decisions, where individuals were deciding between two products in a category (e.g., types of bandages). These findings suggest that preferring complex information may lead to different decisions when provided with it. However, in many daily choices we face a more complicated task as we must also decide which causal path to intervene upon (e.g., whether to modify diet or activity) rather than which items in a category to select (e.g., which flavor of cream cheese to put on a bagel).

While people may prefer a comprehensive model that more fully accounts for outcomes, it is not yet known whether people can successfully use complex models in decision making. Without any way to easily chunk information in a diagram (van Merriënboer and Sweller, 2005), prior work suggests the diagram may be hard to learn from. Further, Bastardi and Shafir (1998) showed that people seek out information that may end up negatively impacting their decisions, though this work did not examine causal information. In short, individuals may find complex models satisfying and may seek out detailed causal information, but it is unknown whether people’s preferences align with the information that will lead better decisions.

Prior work on causal reasoning has primarily focused on people’s ability to learn and make inferences from small causal structures (Rottman and Hastie, 2014) and in scenarios unrelated to prior experience and knowledge. For example, Stephan et al. (2021) examined how people revise their beliefs about causal chains as they get new information (e.g., learning first that $A \rightarrow B$ and later that *C* fully mediates this effect). Research has examined how people use the causal structures they learn to make decisions (e.g., learning which fertilizer makes plants bloom and then deciding what fertilizer to use in a series of sequential trials (Nichols and Danks, 2007)), how people use these decisions to learn causal structures (Hagmayer and Meder, 2008, 2013; Meder et al., 2010), and more broadly how causal Bayesian networks can model people’s reasoning about conditional statements (Oaksford and Chater, 2017). This prior research has focused on novel causal relationships with which participants had no familiarity. It remains an open question as to how people learn when *A*, *B*, and *C* are variables they have existing beliefs about and experience with, such as mental health, exercise, and stress. This is particularly relevant in the context of daily choices where people have a range of existing knowledge and where one must determine whether to provide information comprehensively or in more digestible chunks.

In our work, we focus on decision making on familiar topics, such as health and personal finance, where people bring their own expectations and beliefs (whether right or wrong) to the problem. Rettinger and Hastie (2001) showed that the same decision problem led to different decision outcomes and strategies depending on the domain of the cover story’s topic (e.g., traffic ticket versus stock investment). These findings suggest that prior beliefs and experiences shape how people use information to make a decision. Because people have existing beliefs, the way they evaluate and use causal knowledge may differ significantly from choices made about topics with which they have no experience. A simple causal model of a novel phenomenon may seem complete, because people do not have expectations for what information could be included. On the other hand, a causal model on a topic with which people are familiar may be considered incomplete if people perceive a gap based on their own knowledge. This phenomenon is distinct from complexity: A simple topic with a simple model will still be considered lacking if an individual has beliefs that are not reflected in the model. Further, the information people expect to see may be absent because it is incorrect (e.g., vitamin C curing colds). Thus, it is vitally important to study decisions in familiar domains, where such expectations may play a role.

Recently, we showed that people use causal models in familiar scenarios (e.g., maintaining weight) differently than in novel ones (e.g., mind-reading aliens) (Zheng et al., 2020). We found that giving people relatively simple causal models led to worse decisions than when people are provided with no information and rely on what they already know. This effect was observed only when the questions were about familiar topics, rather than novel scenarios. It remains an open question as to why exactly causal models lead to worse decisions and why this occurs in familiar domains. One possibility is that we tested models that were relatively simple but still applicable to multiple decisions. It may be difficult to integrate causal models with prior knowledge or understand which aspects of a model are most important for a choice if the models provide more information than needed for a given decision. Alternatively, based on work on completeness and complexity, the fact that the models used in previous research were *not* complete, complex, models could be the source of the challenge, as simple models may leave out details people expect to see.

We aim to advance understanding of when and which causal models are useful for decision making. If we provide a complex comprehensive model that captures more causes, will people be able to use it? Alternatively, if we provide a simpler model but make it directly relevant to the question at hand, without any broader causal information, will this too improve decisions, or will people ignore this incomplete model? We conduct a series of experiments designed to shed light on the trade-off between simplicity and complexity in decision making with causal models. In Experiment 1, we compare decision making using simple diagrams containing only information relevant to each choice versus a complex diagram that covers information beyond each specific question. We use both a familiar topic that many individuals have experience with (decisions surrounding bodyweight) and an unfamiliar domain (alien dance-off). In Experiment 2, we expand this to a set of 12 topics covering health, personal finance, life choices, and societal issues, to investigate how effects generalize beyond the domain of Experiment 1. Both Experiments 1 and 2 use simple diagrams that are a subset of more complex models. In Experiments 3 and 4, we test the boundaries of our findings for simple diagrams by exploring what constitutes “simplicity” in a diagram. In Experiment 3, we test whether drawing people’s attention to the relevant part of a complex diagram has the same effect as presenting only that component in a simple form. In Experiment 4, we test whether adding a small amount of irrelevant information to simple diagrams will impede their use for decision making. Finally, given research on preferences for complexity, in Experiment 5 we allow participants to choose how much information they would like on a given topic, enabling us to test if people’s preferences are for the information that will actually help them make a choice.

## Experiment 1: keep it simple, stupid?

We first aimed to test how the level of detail in a causal model influences decision accuracy. Previously we found that causal models can lead to worse decisions than no information at all (Kleinberg and Marsh, 2020; Zheng et al., 2020). The models used were designed to replicate the type of information we receive when making everyday choices about health, and that machine learning methods now create. Thus, the models included causal relationships that did not correspond to any of the target answers in the decision-making questions. In this experiment, we tested whether information tailored specifically to each decision being made can lead to better choices than more complete models that cover multiple potential decisions. If individuals are not able to determine what part of a model to use, or are unable to ignore irrelevant information, we suspect that tailored diagrams will improve choices. On the other hand, simple models are incomplete, which could lead to confusion if information that participants expect to see is missing. In this first experiment, we focused on decision making around bodyweight, as it is a complex topic about which people receive much information and make many decisions.

### Method

#### Participants

We recruited 300 US residents aged 18–64 from Prolific, of which 299 completed the study. Participants were compensated $3.00 based on an estimated study duration of 20 min. We excluded participants who failed our attention check or submitted unusual or duplicate responses (*n* = 36). Thus, 263 participants remain in analysis.

#### Materials

**Fig. 2:**
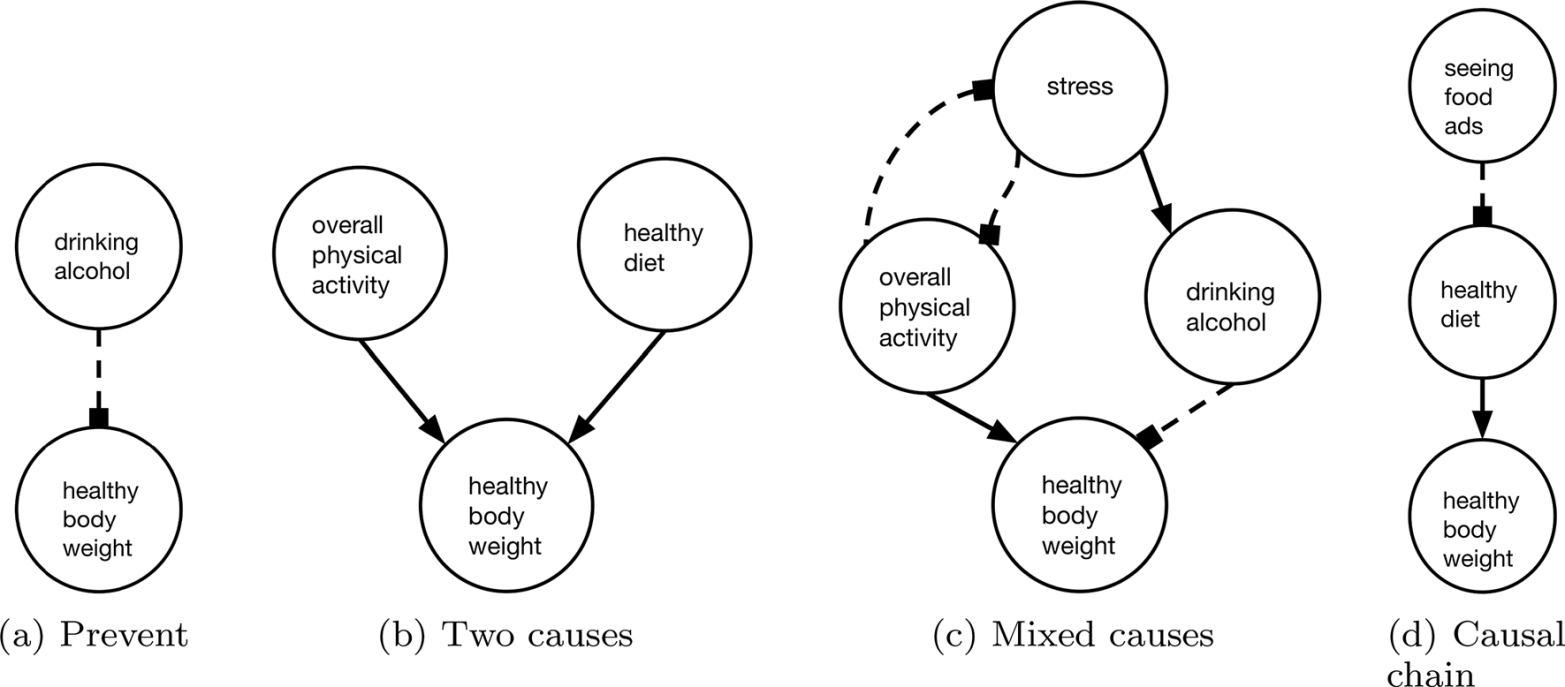
Simple diagrams used in Experiment 1

We created a set of simple causal diagrams that each targeted a different causal process that can influence bodyweight. Using the complex diagram in Fig. 1 as a starting point, each tailored diagram was a subset of the complex diagram. We selected four pathways representing different types of causal structures, creating simple diagrams with only those paths as shown in Fig. 2: (a) a direct preventative relationship (prevent), (b) a common effect structure (two causes), (c) a mix of positive and negative relationships (mixed causes), and (d) a three-node causal chain (causal chain). The first three diagrams (a-c) increase from two to four nodes and require participants to, respectively, select a direct preventative cause, a combination of two positive causes, and to activate a positive cause while deactivating a negative one. Each diagram showed only the causal pathways related to the target answer.^4^

All diagrams use the same format as the original obesity system map, which used solid arrows to indicate generative/positive causes and dashed lines ending in a solid box to indicate preventative/negative causes. In our previous work that found simple diagrams decreased accuracy compared to no information (Zheng et al., 2020), we used plus and minus signs to denote positive and negative causes, respectively. With complex diagrams, where edges may intersect, dashed lines make negative causes more readily apparent.^5^

We created four decision-making questions, one targeting each diagram. The text in these questions described a person who had a goal of managing their bodyweight. For each person, a few sentences provided their life context and any constraints that may exist for their weight loss. The question then presented 4 options of what the person could do to maximize their weight goal. As an example, the question paired with the causal chain diagram was:

Daniel has been overweight for most of his life. He lives with his brother and sister, both of whom also would like to lose weight. They don’t have much free time during the work week, so on weekends they like to relax by watching TV and cooking from new recipes. Daniel is concerned about his weight but doesn’t know what he could do differently.

Which of the following is the BEST suggestion for Daniel?

A. Don’t do anything, weight is genetic
B. Fast forward through TV commercials
C. Get takeout pizza instead of cooking
D. Add more vegetables to his weekend recipes

For questions paired with the prevent, two causes, and mixed causes diagrams, we designated one target answer choice as “correct.” This choice maximized production of the target effect, healthy bodyweight, given the causal diagrams. Other answer choices either represented causal relationships not in the simple or complex diagrams or ineffective relationships that contradicted the diagrams (e.g., an option to increase alcohol intake when the diagram suggests reducing it).

The causal chain diagram differs in that one answer choice corresponded to the direct cause and one to the distal root cause. This enables us to test whether simple diagrams influence how likely participants are to choose a direct cause versus a root node. Causal relationships in health and many other aspects of daily life are rarely deterministic. In a chain of probabilistic causes where there is a single pathway from cause to effect (as in the question used here), intervening on the most direct cause has a higher chance of bringing about the effect compared to intervening on a root node. In this way, the direct cause could be seen as the “correct” answer. Despite this, people have been shown to prefer intervening on root nodes (Hagmayer and Sloman, 2009; Lagnado and Sloman, 2006), suggesting that distal causes could be seen as the correct choice. We analyze responses for this question separately as it is an open question if participants will choose the direct or distal cause, and if diagram complexity influences this choice. The remaining two choices were incorrect in the same ways as the other models’ questions. The full set of questions is included in Appendix 1.

**Fig. 3:**
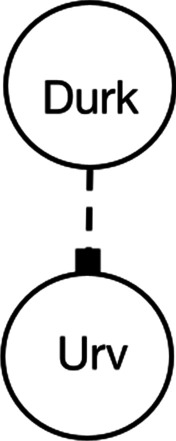
Simple diagram used for direct preventative question about aliens

To test whether findings are specific to domains where people have previous knowledge, we created four novel domain questions that targeted the same causal pathways and use exactly the same complex and tailored causal structures. We replaced the node labels and question text with a scenario participants should presumably not be familiar with, namely an alien dance-off. In the alien dance-off, aliens can cause others to dance faster (positive causal relationship) or dance slower (negative causal relationship). The full set of novel domain questions are included in Appendix 1. The direct preventative question is shown below, and the corresponding simple diagram is shown in Fig. 3. The correct answer is A, as Durk is slowing Urv down.

At the Alien dance off, Durk and Urv are dancing, while Aed and Strin are watching.

How can you make Urv dance faster?

A. Remove Durk from the dance
B. Remove Strin from the dance
C. Make Aed start dancing
D. Make Strin start dancing

#### Procedure

All participants first received instructions on the meaning of causal models and the solid/dashed lines used. Participants were then randomized to an experimental information condition. For the bodyweight questions, participants were assigned to complex diagrams (*n* = 91), simple diagrams (*n* = 88), or no diagrams (*n* = 84). In the complex diagram condition, participants were provided with the full complex diagram seen in Fig. 1. This diagram contains the information needed to answer all presented decision making questions and was presented on the screen with each question. In the simple-diagram condition, participants saw a different diagram for each question that presented only the causal paths from the complex diagram relevant to the question at hand, seen in Fig. 2. In the no diagram condition, participants were not provided with a diagram. This condition served as a baseline for how often the target answers were chosen without the aid of a diagram when people were presumably relying on their previous knowledge. Participants then answered the bodyweight questions by selecting one best option for each question.

After completing the bodyweight questions, participants completed the novel domain questions. They were first instructed on the rules of alien dance-offs. Participants were then assigned to one of two information conditions for the novel questions: complex or simple. We did not include a no diagram condition because the novel stimuli needed an explanation of the causal relationships since there was no prior knowledge to rely on. Participants received a different diagram than what they received in the main experimental questions to make it less obvious that the questions targeted the same pathways. Thus, participants in the simple bodyweight condition received complex novel domain questions, and participants in the complex bodyweight condition received simple novel domain questions. Participants in the no diagram bodyweight condition were randomized to receive either simple or complex diagrams for the novel domain questions.

The order of questions within the experimental and novel domain sections, and order of answers for each question, were randomized for each participant. After completing all decision questions participants completed free-text response questions that presented a diagram with dashed and solid lines connecting alien nodes and asked participants to describe what the diagrams meant. These questions were designed to evaluate participants’ understanding of the novel domain question set up, given the novelty of the decision domain. This further served as a check that participants understood the causal diagrams. We excluded participants who failed this comprehension check.

#### Design and analysis approach

Our one-factor design included information condition as our independent variable and proportion of choosing the target response (i.e., accuracy) as our dependent measure. To calculate accuracy, we calculated for each participant the proportion they chose the target option across the three main questions (prevent, two causes, and mixed causes). We analyzed our data using between-subjects one-way ANOVAs with three levels for bodyweight questions (no diagram, highlighted, complex) and two levels for novel domain questions (highlighted, complex). We used Sidak-corrected *t*-tests to follow-up multiple comparisons.

### Results

#### Effect of diagram complexity on decision accuracy

We first examined results on the novel domain questions, to determine whether participants were able to use our causal diagrams to make decisions with unfamiliar materials. A one-way ANOVA with information condition (simple vs. complex) as a between-subjects factor found a significant main effect (*F*(1, $261) = 27.2, p <.001, \eta _{p}^2 =.094$), indicating that performance was significantly better with a simple diagram ($M =.814$, $\textrm{SE} =.023$) than a complex diagram ($M =.626$, $\textrm{SE} =.028$).

The novel domain questions established that diagrams tailored to the decision at hand can be more useful than a more comprehensive diagram that conveys more information. We next examined whether simple diagrams still have an edge in real-world questions where prior knowledge may interact with the provided information. A one-way ANOVA with information condition (no diagram, simple, complex) as a between-subjects factor found a significant main effect, *F*(2, $$260) = 9.65$$, $$p <.001$$, $$\eta _{p}^2 =.069$$. Performance in the simple condition ($$M =.841$$, $$\textrm{SE} =.030$$) was significantly better than the no diagram ($$M =.683$$, $$\textrm{SE} =.031$$; $$p =.001$$) and the complex ($$M =.681$$, $$\textrm{SE} =.028$$; $$p <.001$$) conditions, while the no diagram and complex conditions did not differ from each other, $$p = 1$$.

#### Effect of diagram complexity on selection of direct causes

We next tested whether diagram complexity influenced choice of direct versus distal causes in the bodyweight questions. We used *N*-1 Chi-squared tests to compare the proportion of participants who chose the direct cause (healthy diet) across conditions. Table 1 presents percentage of people who chose the answer corresponding to a direct cause or a more distal (seeing food ads). Note that the numbers do not sum to 100% as there were four answer choices.

**Table 1:** Proportion (and standard error) of respondents choosing direct or distal causes by information question for the causal chain question in Experiment 1

Question type	Choice	No diagram	Simple	Complex
Bodyweight	Direct	0.96 (0.02)	0.55 (0.05)	0.85 (0.04)
	Distal	0.01 (0.01)	0.44 (0.05)	0.12 (0.03)
Aliens	Direct	–	0.37 (0.04)	0.56 (0.04)
	Distal	–	0.50 (0.04)	0.26 (0.04)

In the no diagram condition, when participants had to rely on their existing knowledge, the vast majority of participants chose the answer representing the most direct intervention. Many participants also chose the direct cause in the complex condition. However, when provided with the simple diagram a smaller proportion of participants chose the direct cause than in the no diagram condition ($\chi ^2(1, N = 172) =$ 40.0, $p <.001$) or complex diagram condition, $\chi ^2(1, N = 179) =$ 19.1, $p <.001$. There was also a significant difference between the no diagram and complex conditions, $\chi ^2(1, N = 175) =$ 6.88, $p =.009$). Thus, when participants were presented with a three-node causal chain, intervening on the most distal (root) cause was a much more popular option than when a complex diagram or no diagram was provided.

We additionally analyzed the preference for direct causes in the corresponding novel domain question. As for the bodyweight questions, the proportion of people choosing the direct cause was significantly lower in the simple than the complex condition, $\chi ^2(1, N = 263) =$ 9.20, $p =$.002. Rather, the distal cause was more popular for the simple condition.

### Discussion

Prior work uncovered a conflict: people are able to learn about and use causal models (Rottman and Hastie, 2014) and yet visual depictions of such models can lead to worse decisions when combined with people’s existing knowledge (Zheng et al., 2020). Our findings begin to resolve this conflict by illuminating where models can help in everyday decisions and what models are most helpful. First, we can reject the hypothesis that simple models impede decisions because they do not provide a full accounting of a causal structure. As our experiments on both real-world (weight management) and novel domain (alien dance-off) questions find, simpler models can outperform complex ones. Second, a critical difference between the simple models used in this experiment and those used in prior work is that our diagrams contain only information needed to successfully answer the question. Thus, this provides a first positive step toward identifying when causal information is useful: causal models can be helpful even when people have prior knowledge if they are restricted to information pertaining to the specific decision at hand.

On our question pitting direct against indirect causes the vast majority of participants selected the direct cause in the no diagram and complex diagram condition, while significantly fewer did so in the simple diagram condition. In our simple chain, the direct cause has a higher probability of leading to the effect. In more complex diagrams, though, a root cause may have multiple paths to an effect, leading to overall greater influence. Thus, neither choice is normatively correct. Rather, the key implication of our findings is the significant impact of presentation on steering people toward more distal causes (which may be undesirable when it is possible to intervene on a direct cause). Prior work has found that people have a preference for intervening on root nodes in causal networks (Hagmayer and Sloman, 2009; Lagnado and Sloman, 2006; Yopchick and Kim, 2009). It appears we can trigger this preference by presenting simple diagrams, where a node that is not normally a root, because it is part of a longer causal chain, appears to be a root cause. Future work is needed to fully understand the mechanism behind this preference. One possibility, given that preferences in the no diagram and complex conditions are very similar, is that the root node is more salient in the simple condition. Given previous work on preferences for root nodes, these may seem like the best option. Another possibility is that a complex diagram may put multiple distal nodes in competition for what is the “most” root cause, driving people to choose a closer cause that has less competition. Future research should test these possibilities.

**Fig. 4:**
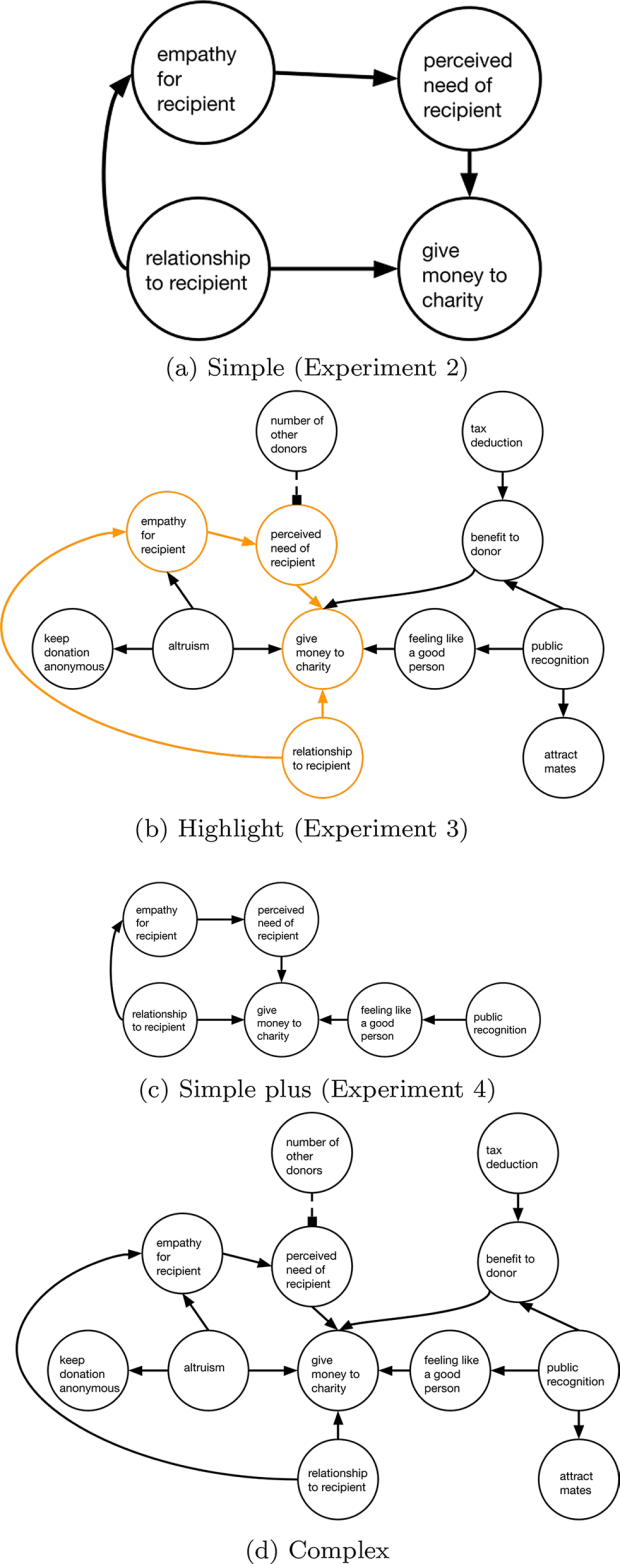
Information conditions used in Experiments 2–5 for the donating to charity question

## Experiment 2: testing other domains

Our first experiment showed that diagrams containing only the information needed to select the correct answer led to better choices. As that experiment used multiple questions about a single topic (managing bodyweight), we now test whether this effect occurs in a wider variety of everyday topics spanning health, personal finance, life decisions, and societal issues.

### Method

#### Participants

As in Experiment 1, we recruited 300 U.S. residents aged 18–64 from Prolific who were compensated $3.00. No participants provided duplicate or unusual answers that would lead to exclusion.

#### Materials

Using a similar approach as in Experiment 1, we now expand to a variety of domains. We selected four domains about which individuals make choices and 3 topics within each domain that we can expect people to feel familiar with: personal finance (saving money, lowering income taxes, planning for retirement), health (mental health, COVID-19, childhood obesity), life decisions (picking a college, buying a house, job satisfaction), and societal choices (donating to charity, carbon footprint, deciding to vote). These are situations about which people commonly make choices and for which they receive guidance, and include decisions made for ones self (e.g., saving money) and choices involving others (e.g., childhood obesity).

For each topic, we used published guidance to create a complex diagram with 12 variables (nodes). We varied complexity so that for each of the four domains there were diagrams with 12, 14, and 16 causal links (edges). All of the 16-edge diagrams included one feedback loop. We systematically varied the target causal pathways our questions probed so they would target different causal structures, ensuring the questions would not seem repetitive. The target structures used were: a two-link chain (2), two direct causes (4), two causes where one or both are indirect (4), and a single cause with two paths to the effect (2). Table 2 shows the topics and model features for each diagram. As complex diagrams all had the same number of nodes, we list only the number of edges in the table, followed by the subset of the structure that was used in the simple diagram. An example simple diagram for the charitable giving question is shown in Fig. 4a. The corresponding question is shown below, and the full set of questions and diagrams are given in Appendix 1.

Erica has been out of work for a few months. It’s been hard on her, but her cat Lucky has been by her side. Lately Lucky has had trouble eating. The vet says he needs expensive dental surgery that Erica cannot afford. Erica feels bad asking people for help, but wants Lucky to get the care he needs. She has never used GoFundMe before but she plans to make a page so people can donate and help Lucky.

What is the BEST suggestion for Erica to make her fundraising successful?

A. Set a smaller goal to start
B. Send the GoFundMe link to close friends and family first
C. Make all donations anonymous but send thank you notes
D. Let people know her cat is still in good spirits

**Table 2:** Domains, individual items, and diagram characteristics for Experiments 2–5

Domain	Item	Complex edges	Simple structure
Finance	Income taxes	12	Two causal paths (one 3-node chain, one direct cause)
Finance	Saving money	14	Two direct causes
Finance	Saving for retirement	16	One cause with two paths
Health	Childhood obesity	12	Two indirect causes
Health	Mental health	14	Two causal paths (one 3-node chain, one direct cause)
Health	COVID-19	16	Two direct causes
Life choices	Buying a house	12	Causal chain
Life choices	Job satisfaction	14	Two causal paths (one 3-node chain, one direct cause)
Life choices	Picking a college	16	Two direct causes
Society	Carbon footprint	12	Causal chain
Society	Donating to charity	14	One cause with two paths
Society	Getting people to vote	16	Two direct causes

All Complex diagrams had 12 nodes

#### Procedure

Participants were randomized to one of three conditions for all twelve topics: simple diagram (*n* = 103), complex diagram (*n* = 96), or no diagram (*n* = 101). As there were twelve questions, we did not use the novel domain questions about aliens or the corresponding alien attention check to keep the study duration roughly the same as in Experiment 1. All other procedures were the same as Experiment 1.

#### Design and analysis approach

Our one-factor design used information condition as our between-subjects independent variable and accuracy as our dependent measure. Given that different topics could have different baseline levels of accuracy, we did not collapse across questions to calculate accuracy as in Experiment 1. Instead, we used a generalized linear mixed model (GLMM) where each topic was a separate repeated measure, with responses coded as 0 when participants did not choose the target (incorrect) or 1 when they did (correct). We used a binary logistic link function to statistically model accuracy as a repeated-measures dependent variable. We entered information condition (no diagram, simple, complex) as a fixed effect into the model. We included random intercepts at both the participant and topic level. This allowed us to test the influence of information condition above and beyond any participant-level or topic-level differences in decision accuracy. The model was run using the Laplace approximation through the PROC GLIMMIX function of SAS (v. 9.04.01). We conducted Tukey-corrected follow-up *t*-tests to compare between specific levels of our factor of interest. We present the estimated marginal means from the GLMM model, transformed back into the probability metric of our rating scale.

### Results

Our GLMM analysis found a significant effect of information condition, *F*(2, $$3289) = 7.98$$, $$p <.001$$. Follow-up pairwise comparisons found that the accuracy in the simple condition ($$M =.725$$, $$\textrm{SE} =.043$$) was significantly higher than in the complex ($$M =.604$$, $$\textrm{SE} =.052$$; $$p =.0011$$) and no diagram ($$M =.590$$, $$\textrm{SE} =.052$$; $$p <.001$$) conditions. As in Experiment 1, we did not find a significant difference between complex and no diagram conditions, $$p =.723$$.

### Discussion

We expand on the findings of Experiment 1, showing that causal models tailored to a decision led to better choices even when testing across a wide variety of topics spanning four familiar domains. This finding is significant given the nature of guidelines given to decision makers. Guidance is often meant to apply to multiple decisions (e.g., news articles on saving for retirement, tips from government health organizations) as opposed to being tailored for one decision. Further, all computational methods for learning causes from data have a similar goal: learn what relationships hold in that dataset. When given to a user, this computationally generated model is not necessarily specific to any given decision and may support many decisions. Our findings suggest that people may have difficulty using these models many data-driven methods produce.

One way that complex, computationally generated, models may still be useful is if attention can be focused on relevant parts of a complex model. Given prior work on people’s preferences (Lim and Oppenheimer, 2020) individuals may resist receiving focused information versus more complete information when they have a choice. As complicated models are often produced and people may prefer them, it is important to learn whether directing people’s attention to the information needed for a decision within a complex diagram can match the benefits of simple models.

## Experiment 3: highlighting what matters

Experiments 1 and 2 found that showing participants subsets of the complex diagram with only the relevant paths for each decision led to better decision accuracy. Instead of showing only the paths needed to select the right choice, we next tested whether directing people’s attention to these paths within a more complex model is sufficient for obtaining the benefits of tailored information. In Experiment 3a, we tested this with the bodyweight and novel domain questions of Experiment 1, and in Experiment 3b we used the same procedure with the 12 topics from Experiment 2.

### Experiment 3a: bodyweight

#### Method

**Participants** We recruited 300 US residents aged 18–24 on Prolific, with 290 completing the study. Participants were compensated $3.00. As in Experiment 1, we excluded participants who failed our attention check or submitted nonsense or duplicate answers (*n* = 37). A total of 253 participants remain in analysis.

*Materials* We use the same protocol and materials as in Experiment 1, with the difference being that instead of simple diagrams, we highlighted the nodes and edges from our simple diagrams within the complex diagram. For example, instead of seeing the prevention diagram of Fig. 2a, participants saw those nodes and edges depicted in orange in the complex diagram as in Fig. 4b. Orange was chosen to grab attention without prompting any associations with positive or negative effects. Thus, participants in the highlighted diagram condition (highlight) see different parts of the complex diagram highlighted for each question, while the no diagram and complex diagram groups remain the same as in Experiment 1. We follow the same approach for the novel domain questions.

*Procedure* The procedure was the same as Experiment 1, with the addition of instructions of the meaning of the highlighting. Specifically, after the other instructions we added the text “Some figures may highlight information that is most important for the question. This will be shown in orange, like in the figure below.” Below this text was a small diagram showing what the highlighting would look like.

*Design and analysis approach* Our one-factor design used information condition as a between-subjects independent variable. As in Experiment 1, we calculated our dependent variable of accuracy as the mean proportion of choosing the target answer across the three main questions for novel and bodyweight questions separately. We analyzed our data using between-subjects one-way ANOVAs with three levels for bodyweight questions (no diagram, highlighted, complex) and two levels for novel domain questions (highlighted, complex). We used Sidak-corrected follow-up tests for all comparisons.

#### Results

*Effect of highlighted causal paths on decision accuracy* Our hypothesis was that highlighting would have a similar effect to the simple diagram. For the bodyweight questions, we found a main effect of information condition, *F*(2, $$250) = 5.19$$, $$p =.006$$, $$\eta _{p}^2 =.040$$. Performance in the highlighted condition ($$M =.757$$, $$\textrm{SE} =.033$$) was marginally but not significantly better than the no diagram condition ($$M =.654$$, $$\textrm{SE} =.031$$; $$p =.079$$) and was significantly better than the complex condition ($$M =.615$$, $$\textrm{SE} =.034$$; $$p =.006$$). Once again no diagram and complex conditions did not differ from each other, $$p =.778$$. Overall, the pattern of means replicates Experiment 1 and suggests that focusing attention on the relevant aspects of a complex diagram provides some of the benefits of simple information while allowing the complete model to be shared.

For the novel domain questions, we collapsed all participants who saw the highlighted version of the diagram into one group and similarly all participants who saw the complex diagram without highlighting into another group. We did not find a significant main effect of information condition, $p =.157$, indicating that performance was not significantly better with the highlighted novel domain diagram ($M =.620$, $\textrm{SE} =.0263$) compared to the complex diagram ($M =.562$, $\textrm{SE} =.029$).

**Table 3:** Proportion (and standard error) of respondents choosing direct or distal causes by information question for the causal chain question in Experiment 3a

Question type	Choice	No diagram	Highlighted	Complex
Bodyweight	Direct	0.93 (0.03)	0.64 (0.05)	0.80 (0.04)
	Distal	0.03 (0.02)	0.33 (0.05)	0.13 (0.04)
Aliens	Direct	–	0.48 (0.04)	0.52 (0.05)
	Distal	–	0.28 (0.04)	0.24 (0.04)

*Effect of highlighted paths on preference for direct causes* In Experiment 1, we found that a simplified diagram increased the proportion of people choosing distal causes. We tested whether this holds when highlighting the same paths within a complex diagram, and present results in Table 3. As in Experiment 1, most participants in the no diagram and complex diagram conditions chose the answer corresponding to the direct cause compared to the distal cause. However, this proportion is significantly reduced in the highlighted condition. Comparing the frequency with which participants chose the direct cause across conditions using *N*-1 Chi-square tests as in Experiment 1, we found that more participants chose the direct cause in the complex than the highlighted condition ($\chi ^2(1, N = 172) =$ 5.03, $p=$.025), and more chose the direct cause in the no diagram than the highlighted condition ($\chi ^2(1, N = 170) =$ 19.86, $p <.001$). More people chose the direct cause in the no diagram than the complex condition ($\chi ^2(1, N = 164)=$ 5.80, $p=$.016). Thus, once again the direct cause was less popular when attention is drawn to its parent.

In the novel domain questions, we did not see this pattern. Instead, similar percentages of participants chose the direct cause across both conditions, $p=.579$.

### Experiment 3b: varied domains

We next tested the influence of highlighting on decisions in a broader set of domains.

#### Method

*Participants* We recruited 300 US residents aged 18–24 on Prolific, with all completing the study. Participants were compensated $3.00. No participants provided duplicate or unusual answers that would lead to exclusion.

*Materials* As in Experiment 3a, we created versions of each complex diagram from Experiment 2 where the paths from the simple diagrams for each question are depicted in orange.

*Procedure* The procedure was the same as Experiment 2, with the addition of instructions of the meaning of the highlighting.

*Design and analysis approach* Our one-factor design used information condition as a between-subjects independent variable and accuracy as our dependent variable. We used the same GLMM approach as in Experiment 2, with information condition (no diagram, highlighted, complex) as a fixed effect, and random intercepts of participant and topic entered into the model. We used Tukey-corrected follow-up *t*-tests to compare between levels of our factor of interest.

#### Results

We found a significant main effect, *F*(2, $$3289) = 14.8$$, $$p <.001$$. We found the same pattern of means as in Experiment 3a, with participants having higher mean accuracy in the highlighted condition (*M* = 0.811, $$\textrm{SE} =.034$$) than in the no diagram (*M* = 0.658, $$\textrm{SE} =.049$$; $$p <.001$$) and complex diagram (*M* = 0.651, $$\textrm{SE} =.050$$; $$p <.001$$) conditions. There was no difference between the no diagram and complex diagram conditions, *p* =.982.

### Discussion

Our experiments highlighting the correct paths provide support for a second way to improve decisions with causal models. Highlighting the relevant causal paths elicits comparable decision-making behavior as providing only those paths as in the simple diagrams of previous experiments. Thus, we now identify two ways of making causal diagrams useful and usable, both centered on focusing attention: (1) presenting only relevant information and (2) highlighting what is relevant. Further, we see evidence for the same effect of Experiment 1, that people showed less preference for direct causes in the highlighted condition than in the no diagram and complex diagram conditions. Interestingly, we do not find a significant difference between the novel domain complex and highlighted conditions of Experiment 3a. It is an open question for future work to determine why highlighting complex diagrams in an unfamiliar domain may be less likely to make people focus on distal causes.

While prior work did not explore why causal diagrams may impede decisions, the findings of Experiments 1–3 suggest a possible mechanism: the detrimental effect of causal diagrams could be due to the inclusion of information beyond that strictly needed to answer the question. We test this possibility in Experiment 4.

## Experiment 4: can people handle a dash of truth?

Our first three experiments demonstrate that providing diagrams that focus solely on the information that is needed for a decision can lead to better choices than no diagram or complex diagrams. We hypothesized that this is due to removing or de-emphasizing information that is not needed to make the correct choice. To test this, we expanded our simple diagrams by adding a few extra nodes and edges (that do not change the target answer) to see if people can handle a dash of truth or if the dose makes the poison.

### Method

#### Participants

We recruited 400 US residents aged 18–24 on Prolific, with all completing the study without nonsense responding. Participants were compensated $3.00. Sample size was increased compared to Experiments 1–3 to maintain 100 participants per condition with four study conditions.

#### Materials

We used the 12 topics of Experiments 2 and 3b for our target materials. For each of the simple diagrams, we created a “simple plus” version that adds two nodes and edges from the complex diagram. As noted in Experiment 1, when developing answer choices we specifically included some that were contradicted by causal relationships in the complex diagram. Including information that is contradictory or unrelated to the target causal path in the simple plus diagrams should help rule out one of the answers, directing participants even more strongly to the target response. As an example, Fig. 4c shows the simple plus version of the diagram for the charitable giving question, which adds a path that contradicts an answer (e.g., diagram indicates public recognition has a positive effect, but one answer choice is to avoid public recognition and give private thanks).

#### Procedure

The study procedure was identical to that of Experiment 2, with the addition of the simple-plus condition. Participants were randomized to one of four conditions: no diagram (*n* = 100), simple diagram (*n* = 98), simple-plus diagram (*n* = 103), and complex diagram (*n* = 99).

#### Design and analysis approach

Our design was again a one-factor between-subjects design with information condition as our independent variable of interest and accuracy as our dependent measure. As in Experiments 2 and 3b, we used a GLMM with information condition (no diagram, simple, simple plus, complex) entered as a fixed effect and the random intercepts of participant and topic entered into the model, with Tukey-corrected *t*-tests to compare between conditions.

### Results

We found a significant main effect of information condition, *F*(3, $$4389) = 10.6$$, $$p <.001$$. As in Experiment 2, participants in the simple diagram condition had higher mean accuracy (*M* = 0.746, $$\textrm{SE}$$ =.035) than participants in the no diagram (*M* = 0.540, $$\textrm{SE}$$ =.045 $$p <.001$$) or complex diagram (*M* = 0.586, $$\textrm{SE}$$ =.044; *p* =.001) conditions. In addition, seeing only a small amount of extra information (simple plus: *M* = 0.633, $$\textrm{SE}$$ =.042) significantly reduced accuracy compared to the simple condition, *p* =.009, while accuracy in the simple plus, no diagram, and complex diagram conditions did not significantly differ from each other, *p*s $$>.08$$.

### Discussion

This experiment tested the boundaries of the effects seen in Experiments 1 and 2, examining whether diagrams that previously aided participants would have the same effect with the addition of a small amount of extra information. We again replicated that a simple diagram, restricted only to the information needed for the question at hand, can aid in decision making. However, the addition of a small amount of information not directly related to the target causal path in the simple-plus condition led to a sharp decline in selecting the target choice. Adding in only two additional nodes and edges led to accuracy on par with that of the complex diagram. This suggests that a model’s overall complexity is not the primary factor leading to inferior decisions with causal models. Rather, any information not directly needed to make the target choice can be distracting and lead to a negative effect.

Prior work has shown people often prefer complex explanations of complex phenomena (Lim and Oppenheimer, 2020; Zemla et al., 2017). Since all of our topics involve complex, real-world, decision making, this would suggest that incorporating more information may make the diagrams closer to what people prefer. It is possible, though, that people may differ in how much information they would like to have about a given domain and would have performed better if they were able to choose what information to receive. That is, we do not know whether the particular individuals who would prefer a complex model would be better able to use them, or whether there is a disconnect between the information people think they want and the information they really need.

## Experiment 5: pick your poison

In our first four experiments, individuals were randomized to the various information conditions (no diagram, simple, complex, highlighted, and simple plus diagrams). Prior work has suggested that people may prefer different levels of complexity based on the topic at hand (Lim and Oppenheimer, 2020) and that within a topic people may vary in how much information they desire (Fernbach et al., 2013). This work has primarily examined people’s preferences and the types of information they find credible or satisfying. While detailed information may be appealing, it does not mean that people are able to successfully use it when it is time to make a decision.^6^ We examined whether people are able to determine what information they actually need. By allowing individuals to choose how much information they want for each topic, we tested whether a restricted diagram still outperformed all other conditions, or if individuals have insight into when they would benefit from complex information and when they do not need any information at all.

### Method

#### Participants

We recruited 800 US residents aged 18–24 on Prolific, with all completing the study without duplicates or issue. Participants were compensated $4.50, as we expected a longer (30 min) study duration. Sample size was doubled from Experiment 4 as we expected the distribution of choices would not be uniform and we aimed to ensure sufficient sample size for each information condition selected.

#### Materials

We used the same study questions as in Experiment 4. At the end of the study, we added two questions to assess participants’ experience with each topic. We asked “For each of the following topics please rate how familiar you are with making decisions about it.” and “How confident would you feel making a decision about each of the following topics?” for all twelve decision topics. Participants rated how familiar/confident they were for each topic from 1 (not at all) to 7 (extremely).

#### Procedure

The same basic procedure was used as in Experiment 4 with the key difference being that immediately before each question participants were shown the topic (i.e., ‘Your next question will be related to donating to charity.”) and asked how much information they would like. The four options corresponded to our four conditions: I don’t need any information (no diagram), Give me just the basics (simple diagram), Give me the basics plus a little extra (simple plus diagram), and Give me all the information (complex diagram). Prior to the twelve decision-making questions participants saw instructions about the meaning of causal diagrams and saw a sample question on bodyweight (which was not a topic in the main experiment) along with an example of what each information choice would look like for that question. The order of the 12 topics was randomized for each participant, and before each question participants made an information choice for it. This allowed participants to select diagrams providing more information for some topics and less for others. Following the decision questions, participants completed a demographic questionnaire and the familiarity/confidence ratings.

#### Design and analysis approach

Our one-factor design tested the effect of our independent variable of interest (information choice: no diagram, simple, simple plus, complex) on our dependent variable of accuracy. We used the same GLMM analysis strategy as in Experiment 4. Our base GLMM included information choice (no diagram, simple, simple plus, complex) as a fixed effect, and random intercepts at the participant and topic level. Additional analyses described after our main analysis added more variables as fixed effects. We used follow-up Tukey-corrected *t*-tests to compare between levels.

### Results and discussion

#### Information choices and effects

First, we averaged across participants to determine what diagrams were most often selected across topics. The most popular information selection was the simple diagram (42% of selections across topics), but many participants opted for no information (21%), simple plus (23%), and to a lesser extent complex diagrams (14%).

We next examined how information choices influenced decision accuracy. We found a main effect of information choice, $F(3, 8786) = 37.1, p <.001$. Simple diagrams led to higher accuracy ($M =.802, \textrm{SE} =.029$) than no diagram ($M =.646, \textrm{SE} =.043$; $p <.001$) and complex diagrams ($M =.697, \textrm{SE} =.041$; $p <.001$). The difference between simple and simple-plus diagrams ($M =.770, \textrm{SE} =.033$) did not reach significance with Tukey correction, $p =.0823$. Accuracy with simple-plus diagrams was significantly higher than when no diagram or complex diagrams were selected, *p*s $<.002$. There was no difference in accuracy when complex diagrams or no diagram were chosen, $p =.129$.

#### Exploration of other influences on information choices and decisions

Given the large sample size used in this experiment, we next explored how other factors we measured could influence accuracy above and beyond diagram choice. Prior work suggests that confidence is a salient aspect of decision making (Yeung and Summerfield, 2012), so we first explored the relation of confidence and familiarity to accuracy. We calculated mean confidence, familiarity, and accuracy for each participant by averaging across all topics for each measure. These mean values give a sense of participants’ overall confidence, familiarity, and accuracy across topics. We then calculated Pearson’s correlations to test the relation between these overall values. We found that familiarity and confidence were significantly positively correlated, $r(758)=.80$, $p <.001$, while accuracy was negatively correlated with both familiarity ($r(758)= -.09$, *p* =.010) and confidence, $r(758)= -.15$, $p <.001$.

Our finding of a negative relationship between confidence and accuracy is interesting given previous literature that suggests a positive link between confidence and accuracy (Yeung and Summerfield, 2012). At the same time, people are regularly found to lack insight into the extent of their knowledge (Rozenblit and Keil, 2002). Thus, we investigated the relationship between confidence, diagram choice, and accuracy. Descriptively, we found that mean confidence was highest when no diagram was chosen ($M = 4.92, \textrm{SE} = 0.057$) than when any other diagram choice was made (simple: $M = 4.74, \textrm{SE} = 0.044$; simple plus: $M = 4.62, \textrm{SE} = 0.056$; complex: $M = 4.46, \textrm{SE} = 0.080$).

We next examined whether confidence can account for the main effect of diagram choice on accuracy we found in previous analyses. We used the same basic GLMM analysis as previously but now entered information choice (no diagram, simple, simple plus, complex) and confidence as fixed effects. As before, we entered the random intercepts of participant and topic. We found a main effect of information chosen, $F(3, 8785) = 36.6, p <.001$. The main effect of confidence did not reach significance, $p =.0620$.

**Table 4:** Results of Experiment 5 by demographic groups. Numbers indicate average accuracy across topics when members of the group selected that level of information

	Gender		Education		
	Woman	Man	HS or less	BA or BS	Grad school
No info	0.64	0.59	0.61	0.62	0.61
Simple	0.80	0.73	0.78	0.79	0.65
Simple plus	0.76	0.69	0.74	0.77	0.63
Complex	0.69	0.59	0.67	0.64	0.54

A possible reason why confidence did not have a significant main effect in this analysis is that confidence may be part of the variability accounted for by the random intercept of topic. Baseline differences in accuracy by topic may come from an array of factors, including complexity of the domain or frequency with which such decisions are made. If confidence is a component of the baseline differences in accuracy by topic, then there may not be much room for confidence to predict accuracy above and beyond what is accounted for by topic. To provide evidence for this account, we reran the GLMM but left the random intercept of topic out of the model. We found a significant main effect of confidence, $F(1, 8796) = 19.9, p <.001$ and a significant main effect of information chosen, $F(3, 8796) = 32.9, p <.001$.

We took this same statistical approach to explore the relationship of familiarity and accuracy. We found a main effect of information chosen, $F(3, 8785) = 36.9, p <.001$, but no main effect of familiarity, $p =.267$, when participant and topic were included as random intercepts. When topic was removed from the model, the main effect of familiarity was significant, $F(1, 8796) = 23.4, p <.001$, as was the main effect of information chosen, $F(3, 8796) = 33.0, p <.001$. These findings highlight how confidence and familiarity are components of baseline differences in accuracy across topics. Importantly, both confidence and familiarity ratings were elicited after the decision-making task. Thus, these ratings could have been influenced by perceptions of the difficulty of the decision-making questions.

Given our findings on confidence and familiarity, we next explored whether other factors linked to confidence were related to accuracy on our decision-making questions. Differences in decision-making confidence have been linked to both gender (Bleidorn et al., 2016; Estes and Hosseini, 1988) and education (Bhandari and Deaves, 2006). We examined whether gender and education separately were related to accuracy.^7^ In Table 4, we present the mean accuracy for each information choice by gender and education.

We ran the GLMM analysis with information choice and gender (man vs. woman) as fixed effects, including the random intercepts of participant and topic. We also included the interaction of information choice and gender because we are interested in whether any potential gender differences in accuracy vary depending on which diagrams were selected. We found a significant main effect of information choice, $F(3, 8585) = 36.5, p <.001$, and of gender, $F(1, 8585) = 22.89, p <.001$. There was no interaction of gender and information choice, $p =.151$. Exploring the main effect of gender, we found that women ($M =.776, \textrm{SE} =.0325$) were more likely to choose the target answer than men ($M =.693, \textrm{SE} =.039$). Given that there was not a significant interaction, this pattern for accuracy did not differ depending on which information choice was made.

From our analyses, it is not clear why gender is related to accuracy. As we described previously, baseline accuracy differences across topic likely reflect differences in the inherent familiarity and confidence participants have with the topic, among other factors. Future work exploring accuracy differences across domains may also examine how variables influencing baseline accuracy may cluster by gender.

We next looked at education level. People with higher levels of formal education may be more accustomed to reading the type of diagrams we used to represent causality, resulting in better use of those diagrams. We collapsed education completed into three groups: high school or less ($n = 256$), bachelor’s ($n = 355$), and some form of graduate education ($n = 169$).^8^ We entered information choice and education level as fixed factors in our model, along with the random intercepts of participant and topic. We also included the interaction of information choice and education. We found a significant main effect of information choice, $F(3, 8560) = 27.4, p <.001$, and of education, $F(2, 8560) = 12.43, p <.001$. There was a significant interaction of education and information choice, $F(6, 8560) = 2.47, p =.0217$. Participants who reported some form of graduate education had lower accuracy than the high school or less and the college completion groups when selecting simple and simple-plus diagrams, *p*s $<.045$. The graduate education group did not differ in accuracy from the other two groups when no information or complex diagrams were chosen, *p*s $>.85$. There was no difference between the high school or less and college groups in accuracy for any of the diagram choices, *p*s $>.98$. These results suggest that the effects of education are driven by highly educated individuals having worse accuracy when using simpler diagrams. Future work is needed to determine the mechanisms behind this effect. It is possible that more highly educated individuals were less trusting of the simple information and may believe there are other important factors that are missing. We have previously found that when expected information is omitted from a causal model, participants trust the model less, while omitting less expected information actually increased trust (Kleinberg et al., 2022). Overall, our findings with demographic variables suggest new avenues for researchers to explore for why certain individuals may have more or less success in making decisions for different topics, and to further tailor diagrams to both decisions and individuals.

## General discussion

Across a set of six experiments, we show that causal models can aid decisions, but that people need help understanding what information in a model is relevant. We previously (Zheng et al., 2020) found that causal models led to worse decisions in familiar domains, and our first experiment here sheds light on a potential mechanism. The key difference between the causal models used in our prior work and in this work was the presence of information that could be used to support multiple decisions. In the current work, our simple models contained solely the causal pathways related to the target answer.

Based on prior literature, there are two likely mechanisms that could underlie our results: difficulty integrating presented information with existing knowledge, and difficulty determining what parts of a causal model to use for a given decision. The integration mechanism would suggest that the reason people perform worse with causal models in familiar settings is that they already have some knowledge about the topic and have difficulty combining this knowledge with the models. For example, a person who believes that multiple diets such as a keto diet or a Mediterranean diet could maintain weight may not be sure which or if either diet corresponds to the node “healthy diet”. Someone else may have beliefs that directly contradict a node (e.g., not believing that alcohol consumption influences weight) or may have additional knowledge not represented in the model (e.g., knowledge of medications that cause weight gain). Under this hypothesis, as the models grow more complex, people should perform worse as there is more information to integrate. Our results in Experiment 3, where we highlighted paths in complex models, provide evidence against this potential mechanism as the primary explanation. The highlighted and complex conditions present the same amount of information, with the only difference being that the highlighted condition draws attention to the part of the complex diagram that is useful for making the decision at hand. If people were only having difficulty integrating their existing knowledge about a topic with the diagram, these two conditions would present the same challenge since they present the same overall information.

On the other hand, if participants do not know which parts of a diagram matter for a given decision, then highlighting would be beneficial as it would cue them on where to focus. That is what we observe in Experiment 3. The simple-plus condition of Experiment 4 further supports this mechanism. In that experiment, we tested what happens when we add a small amount of irrelevant information. The increase of information should increase difficulty but accuracy should be higher than the complex diagram as there is less information to integrate. The models remained relatively simple (and thus likely simple to integrate with existing knowledge), but even a small dose of extra information led to accuracy that was indistinguishable from that of complex causal models or no information at all.

Taken together, we believe our results suggest that the difficulty in using diagrams originates from the difficulty people have in determining what information to use. Further, based on the simple-plus results of Experiment 4 how much extra information is added may be less important than whether it is present at all. Experiment 3 directly addressed this hypothesized explanation by drawing attention to the relevant information. Finding that this improved results compared to complex diagrams, similarly to providing only the relevant information, suggests that knowing what information to focus on is a core challenge to use of causal models to make choices.

One may ask then, if we must block out all information not needed for a choice, are causal models still useful? We note first that simple causal models do lead to greater selection of a target choice than no information, so it does appear that a thoughtfully constructed model can be helpful. However, causal models as they are generally constructed and presented may not be useful or usable in the way their creators envision. While methods for learning causal relationships and models from data usually aim to find the most complete model, and computational methods are frequently evaluated on how well they recover all known causes, the paradox of choice (Iyengar and Lepper, 2000; Schwartz, 2004) gives us reason to suspect that more is not better. More options do not lead to better choices in domains as wide ranging as online dating (D’Angelo and Toma, 2017; Wu and Chiou, 2009) and tourism (Park and Jang, 2013) and can lead to lower satisfaction with choices and people failing to making a choice at all, though effects vary across domains (Scheibehenne et al., 2010). If we consider the paths or each link from cause to effect as a potential option for intervention, a model then provides a set of options for achieving an outcome. As the model grows, it may become demotivating rather than helpful (Iyengar and Lepper, 2000).

Integral to the paradox of choice is that while not leading to the best decisions, people often find having many options appealing (Iyengar and Lepper, 2000; Schwartz, 2004). However, when our participants were able to choose between the different information conditions (Experiment 5), they selected the most complex causal models only 14% of the time. Thus, for our causal diagrams, added complexity that provides a large amount of options to intervene on is not overwhelmingly appealing. Even though it may intuitively make sense to prefer a more comprehensive model that explains more of the world, the model’s users are still humans who have limited cognitive capacity.

A key difference between classic studies that explore the paradox of choice and our work is that all of our questions have a target answer (the one designated as most likely to achieve the stated goal). We find that highlighting (Experiment 3) or presenting only the information that steers people toward that target choice (Experiments 1 and 2) leads them to pick it more frequently. Further, increasing the set of options by a small amount (Experiment 4, simple plus diagrams) significantly reduces people’s likelihood of choosing the target answer. This has important implications for public health agencies, consumer advisors (e.g., for personal finance and housing), and other groups that try to guide decisions.

### Limitations

While our experiments consistently showed that simple information improved decisions compared to complex information and no information, key open questions for future work remain. Based on prior work showing people more likely to make optimal decisions for others versus themselves (Crockett et al., 2014; Zikmund-Fisher et al., 2006), we focused on hypothetical decisions for others. Future work is needed to explore whether causal models differently impact decisions for self versus others. When making a choice for oneself, a decision maker may draw more on their own preferences and context, making them less likely to rely on information in a model. Alternatively, if decision makers still struggle to figure out which part of a model is relevant to their concerns, then complex models may affect self decision-making in the same way as decision making for others. Future work that tailors questions to individual participants and their knowledge could help determine how model use may differ with self versus other decisions.

A second key open problem is moving from hypothetical to actual decisions with consequences. We aimed to test decision making in a wide variety of domains, ranging from decisions that could be made multiple times (e.g., donating to charity) to decisions that may be one-shot major life choices (e.g., choosing a college). This enabled us to evaluate across a range of high and low stakes choices. However, we did not ask people to make a choice they had to enact in a real context, such as opening an actual savings account or ordering a meal. Our results may reflect what people *think* should be done in these situations but not match what they would actually opt for if they had to implement the choice. Field studies that provide participants with causal models alongside making actual purchasing or other similar decisions could help clarify how our results translate into everyday settings. Our research group is specifically investigating how providing causal models of disease in the healthcare setting could facilitate shared decision-making between patients and providers. More generally, it is an interesting question for future research to understand how our findings translate into actual decisions.

Finally, our studies were not designed to determine why people choose one information source over another to aid decision making. In Experiment 5, we saw that people had a bias toward choosing simple diagrams. However, people did not always choose the same type of information across decisions. We have hints in our data that factors such as decision-maker confidence or familiarity can make certain topics more difficult to make decisions in than others. Understanding how these differences by topic translate into people seeking different types of information is an important future direction.

### Conclusion

Guidelines are often presented to the public generically, with information that could support multiple choices (e.g., options targeting diet or exercise to reduce risk of Type 2 diabetes). We find that this may be less effective than presenting information supporting only the best intervention for an individual. Our studies build on the greater literature exploring how causal beliefs guide reasoning and the importance of understanding prior beliefs. Our results suggest that causal information should be given on a need to know basis and that, for the most part, people do not need to know.

## Figures and Tables

**Fig. 5:**
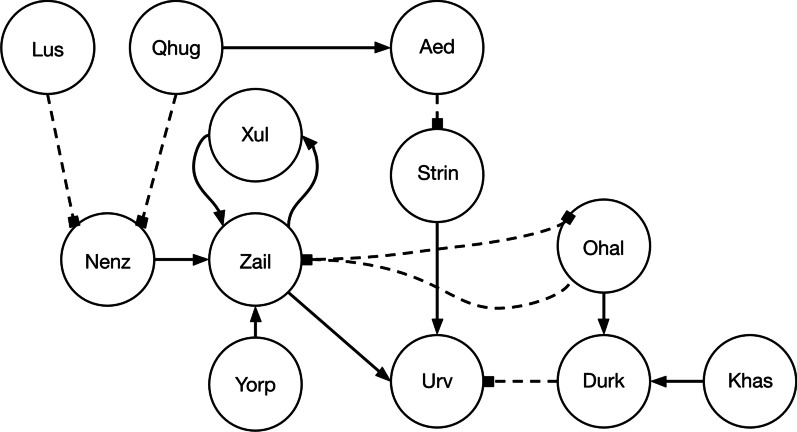
Complex causal diagram on alien dance off structure

**Fig. 6:**
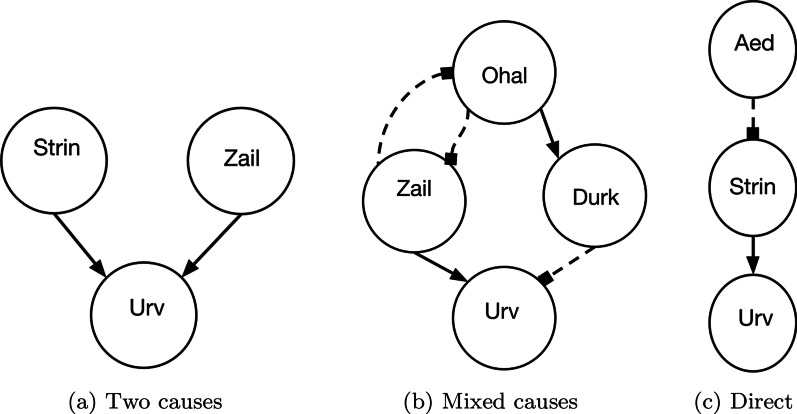
Simple diagrams used in novel domain questions about aliens

## Data Availability

The datasets used and/or analyzed during the current study are available from the corresponding author on reasonable request. The studies were not preregistered.

## Appendix 1

### Experiment 1 materials

#### Direct preventative question

Maria is a recent college graduate who loves to meet up with her friends after work. They usually get hamburgers from a nearby take-out restaurant and enjoy them with a few beers. Lately Maria has noticed her pants fitting a bit tighter. She now wants to make some changes to avoid gaining any more weight.

Which of the following is the BEST choice for Maria to achieve her goal?

A. Get her burger on a gluten free bun
B. Have a diet soda instead of a beer
C. See her friends less
D. Make her own hamburgers

#### Two paths question

Ed and his wife used to love hiking when they first got married, but then he started a construction job that left him too tired to go hiking. He recently started a new job working at a call center where he spends most of his time at a desk. His wife has started baking and Ed looks forward to a dessert at the end of the day. However, he has put on about 30 pounds in the last 6 months.

Which of the following is the BEST thing Ed could do to lose the weight he put on?

A. A few days a week go for a walk with his wife instead of having dessert
B. Try standing more at work
C. Ask his wife to stop baking
D. Stop eating after 8pm

#### Mixed paths question

Sarah has been trying new diets on and off since she was a teenager. None have helped her meet her goals, and she’s found it hard to cook since she’s been stressed out and anxious. Her friend Jane recently tried a Mediterranean diet and liked the results. Sarah wants to make changes but is not sure where to start.

Which of the following is the BEST choice Sarah can make?

A. Follow what worked for Jane, and have avocado and egg for breakfast
B. Skip breakfast
C. Start her day with an online aerobics class and then a calming meditation
D. Have bacon instead of bread for fewer carbs

### Novel domain questions

The complex diagram’s nodes were relabeled with alien names and rotated as shown in Fig. 5.

#### Two paths novel domain question

None of the aliens are dancing.

How can you make Urv dance as fast as possible?

A. Make Strin AND Zail dance
B. Make Strin dance
C. Make Zail Dance
D. Remove Strin from the dance

The corresponding simple diagram is shown in Fig. 6a.

#### Mixed paths novel domain question

At the dance off, Ohal is dancing.

How can you make Urv dance faster?

A. Remove Ohal from the dance AND Make Zail dance
B. Make Durk dance
C. Remove Zail from the dance
D. Remove Ohal from the dance

The corresponding simple diagram is shown in Fig. 6b.

#### Direct novel domain question

At the alien dance off, Urv is the only one dancing.

If you want to make Urv dance as fast as possible, which of these is the best choice?

A. Make Aed dance
B. Make Strin dance
C. Remove Aed from the dance
D. Remove Strin from the dance

The corresponding simple diagram is shown in Fig. 6c.

### Experiment 2 materials

#### COVID-19 question

Linda works at a grocery store, so she has been working at her job in person throughout the COVID-19 pandemic. Her friend Margo has a compromised immune system and has been avoiding leaving home except for doctor’s appointments since she wants to reduce her chance of getting infected. Linda is worried because Margo has complained about how lonely she feels, and Linda wants to help out. Whenever Linda invites her over for dinner though, Margo declines.

What is the BEST suggestion for how Linda can help Margo feel better and stay safe?

A. Drop off a healthy meal for Margo to eat alone
B. Encourage Margo to shop at the grocery store where she works
C. Respect Margo’s preferences and send her a card in the mail

#### Mental health question

Marcus has been working as an investment banker for over a decade. Banking often involves long hours and stressful deadlines, but Marcus says it’s all worth it when he gets a big bonus. In the last few years the hours have increased but his bonuses haven’t. Marcus is now too exhausted to enjoy himself on the weekends and knows he needs to make some changes. However, Marcus just bought a house so he really needs his salary.

What is the BEST suggestion you could give Marcus to help him feel better?

A. Quit his high pressure job
B. Take a walk after work with a friend
C. Have a glass of white wine after work
D. Take a vacation after the next big deadline

#### Childhood obesity question

Candace decided to start homeschooling her son, Joey, about a year ago. Joey is doing much better on his exams and seems a lot happier. Candace spends so much time on homeschooling, though, that she is too tired to cook every night. Without recess and physical education classes, Joey has put on some weight. Candace wants her family to be healthy but doesn’t have much free time.

What is the BEST suggestion you can give Candace to improve Joey’s health?

A. Tell Joey to eat less candy and play video games instead
B. Play soccer with Joey for 15 min after school instead of watching TV
C. Get fast food burgers as a treat only on the weekends
D. Save time by cooking for Joey and getting takeout for herself

#### Buying a house question

Richard lives in a small apartment close to his office. He just adopted a puppy, so he thinks it’s time to buy a house with a yard where the new puppy can play. But Richard is worried that he can’t afford to stay in his neighborhood. There are not many houses for sale in his area, and many parents like the school district his neighborhood is in.

What’s the BEST advice for how Richard can find a house in his budget?

A. Look for houses that need some work
B. Make an offer quickly in case prices go up
C. Look at newly built houses rather than older ones
D. Buy a condo without a yard

#### Job satisfaction question

Diana is a manager at an office supply company. It is hard to keep good employees around, since the company is not in a desirable location and does not allow remote work. Diana’s administrative assistant, Angie, has been on the job for about a year now. She’s the best assistant Diana has ever had. Diana does not have the budget for a raise, so she wonders what she can do to make Angie stay.

What is the BEST way for Diana to improve Angie’s job satisfaction?

A. Implement casual Fridays
B. Tell Angie she would give her a raise if she could
C. Give Angie more responsibility and a new job title
D. Let Angie delegate some tasks to an intern and work flexible hours

#### Picking a college question

Ben works in admissions at a college. The school has strong academic programs, but struggles to attract students due to its rural location. Ben likes the quiet town and hopes to work there for a long time. He worries that if the college cannot attract more students, his job may be in danger. Ben needs to get more students to apply, but needs to make sure they are good students as well.

What is the BEST thing Ben can do to attract more students to his college?

A. Make SAT test scores optional and make books free for students’ first year
B. Include more pictures of the town on the school’s website and do video campus tours
C. Suggest that the school offer fewer majors for students to select from
D. Set a later application deadline than other schools

#### Income taxes question

Nicholas is a freelance writer, so his income varies a lot depending on how many clients he has and when they pay. His wife is a dog walker who has been out of work this year due to having knee surgery. Nicholas just got a new assignment for a magazine. While he won’t get paid till December, the job pays $1000. When he added up all his expected income for the year, Nicholas realized the new job will put him into a higher tax bracket. He expects more income next year when his wife is back at work, so Nicholas is wondering if he can do anything to reduce his taxes.

What is the BEST advice for Nicholas to reduce his taxable income?

A. Ask his clients to pay after January 1st
B. Deduct his wife’s medical expenses and contribute $1000 to a traditional IRA
C. Start filing taxes separately from his wife and contribute $100 to charity
D. Get an extension so he can pay later

#### Saving money question

Gloria is a hairdresser who works at her aunt’s salon. Business was very slow over the summer, so Gloria had to use up some of her savings to pay her rent. She wants to continue paying off her student loans, but also thinks she should rebuild her emergency fund. She enjoys lunch out with her coworkers most days and on the weekends they like to go clubbing together. Gloria wants to save money but never seems to have anything left over.

What’s the BEST way for Gloria to reduce her expenses?

A. Take turns having a potluck at her friends’ houses and have a movie night instead of going out
B. If she has any money left over at the end of the month, save it
C. Check if she’s eligible to defer her student loans
D. Buy wine by the bottle instead of by the glass when she goes out

#### Saving for retirement question

After 30 years as a nurse, Rita is looking forward to retirement. Her job is physically demanding and she does not know how much longer she can do it. Over the years she bought stocks based on tips from friends. But as she gets closer to retirement, Rita gets worried every time the stock market drops. She is thinking of making some changes but is not sure where to start.

What is the BEST way for Rita to reduce her risk?

A. She should not make any changes close to retirement
B. Buy more of her favorite stock each month
C. Sell some of her stocks and buy new ones
D. Keep some stocks, but sell some of her stocks and keep that money in a savings account

#### Carbon footprint question

Tony just welcomed his first child. Tony has never been environmentally conscious, and doesn’t recycle or think much about where the meat he eats comes from. Now that he has a baby, he has been thinking a lot about the future and what the world will be like when the baby grows up. Tony wants to reduce his carbon footprint but does not know where to start.

What is the BEST suggestion for Tony to reduce his carbon footprint?

A. Air dry his laundry
B. Cook at home instead of getting takeout
C. Drive to visit relatives instead of flying
D. Switch to eating locally raised beef

#### Getting people to vote question

Lewis can’t wait to cast his ballot in the upcoming election for mayor. His friend Kim, on the other hand, isn’t sure if she will vote this year. She plans to vote if she has time after work, but her polling place is out of the way and she has a very busy day. Lewis can’t understand why voting isn’t a priority for Kim, and wants to convince her to vote.

What is the BEST thing Lewis can do to get his friend to vote?

A. Tell her that every vote makes a difference since the candidates are currently tied
B. Tell his friend more about the candidates
C. Let his friend know it’s a local election
D. Remind his friend that their preferred party will probably win

### Experiment 2 results

Here we include item-level results per topic.

### Experiment 3 results

The following table provides item-level results for Experiment 3b.

See Tables 5 and 6

**Table 5:** Percentage correct responses by information condition in Experiment 2

Domain	Item	No info	Simple	Complex
Finance	Income taxes	0.54	0.62	0.60
Finance	Saving money	0.64	0.62	0.56
Finance	Saving for retirement	0.71	0.67	0.70
Health	Childhood obesity	0.76	0.83	0.76
Health	Mental health	0.34	0.56	0.51
Health	COVID-19	0.56	0.66	0.43
Life choices	Buying a house	0.73	0.68	0.58
Life choices	Job satisfaction	0.68	0.83	0.69
Life choices	Picking a college	0.44	0.50	0.28
Society	Carbon footprint	0.24	0.59	0.42
Society	Donating to charity	0.57	0.77	0.71
Society	Getting people to vote	0.67	0.79	0.79

**Table 6:** Percentage correct responses by information condition in Experiment 3b

Domain	Item	No info	Highlight	Complex
Finance	Income taxes	0.60	0.78	0.64
Finance	Saving money	0.73	0.78	0.65
Finance	Saving for retirement	0.77	0.69	0.67
Health	Childhood obesity	0.84	0.89	0.83
Health	Mental health	0.35	0.75	0.59
Health	COVID-19	0.61	0.70	0.49
Life choices	Buying a house	0.82	0.77	0.64
Life choices	Job satisfaction	0.88	0.81	0.76
Life choices	Picking a college	0.41	0.61	0.40
Society	Carbon footprint	0.30	0.66	0.46
Society	Donating to charity	0.72	0.78	0.64
Society	Getting people to vote	0.57	0.80	0.67

### Experiment 4 results

The following table provides item-level results for Experiment 4.

See Table 7.

**Table 7:** Percentage correct responses by information condition in Experiment 4

Domain	Item	No info	Simple	Simple plus	Complex
Finance	Income taxes	0.39	0.64	0.58	0.47
Finance	Saving money	0.61	0.67	0.65	0.53
Finance	Saving for retirement	0.69	0.72	0.62	0.77
Health	Childhood obesity	0.64	0.81	0.71	0.67
Health	Mental health	0.25	0.66	0.51	0.51
Health	COVID-19	0.57	0.64	0.59	0.49
Life choices	Buying a house	0.69	0.74	0.60	0.59
Life choices	Job satisfaction	0.68	0.72	0.73	0.63
Life choices	Picking a college	0.47	0.61	0.46	0.43
Society	Carbon footprint	0.26	0.63	0.41	0.40
Society	Donating to charity	0.64	0.73	0.74	0.69
Society	Getting people to vote	0.53	0.76	0.72	0.68

### Experiment 5 results

The following table provides item-level results for Experiment 5. See Table 8.

**Table 8:** Percentage correct responses by information selection in Experiment 5

Domain	Item	No info	Simple	Simple plus	Complex
Finance	Income taxes	0.55	0.74	0.75	0.65
Finance	Saving money	0.66	0.75	0.83	0.64
Finance	Saving for retirement	0.74	0.75	0.75	0.73
Health	Childhood obesity	0.82	0.90	0.89	0.76
Health	Mental health	0.34	0.69	0.67	0.57
Health	COVID-19	0.60	0.68	0.72	0.52
Life choices	Buying a house	0.84	0.82	0.76	0.67
Life choices	Job satisfaction	0.72	0.85	0.85	0.77
Life choices	Picking a college	0.42	0.64	0.48	0.43
Society	Carbon footprint	0.32	0.65	0.53	0.47
Society	Donating to charity	0.66	0.82	0.79	0.63
Society	Getting people to vote	0.65	0.81	0.77	0.68

## Abbreviations

ANOVA: Analysis of variance
GLMM: Generalized linear mixed model
UK: United Kingdom
US: United States

## Footnotes

1 https://www.gov.uk/government/publications/reducing-obesity-obesity-system-map We have adapted the diagram to be suitable for non-medical audiences.

2 https://www.nytimes.com/2010/04/27/world/27powerpoint.html.

3 https://www.jec.senate.gov/public/index.cfm/republicans/2010/8/america-s-new-health-care-system-revealed.

4 We tested a fifth diagram that depicted a feedback loop, but determining a single target answer for the diagram proved difficult, so we do not discuss it further here.

5 To ensure that using dashed lines did not change our original pattern of results, we reran the weight management question of Experiment 1 in (Zheng et al., 2020) with the solid and dashed line format. We replicated the earlier finding, namely decreased accuracy with the diagram compared to no diagram. It does not appear that this presentation style influences accuracy.

6 A parallel idea can be found in the consumer decision-making literature where more choices in an option set can seem appealing, but result in less satisfaction with purchases (Iyengar and Lepper, 2000)

7 We restrict our analyses to participants who self-identified their gender as men ($n = 415$) or women ($n = 367$) because of the small number of non-binary and other responses ($n = 17$).

8 An additional 20 subjects reported an education level that did not fit into one of these three categories (e.g., associate’s degree).
